# The impact of experimental pain on shoulder movement during an arm elevated reaching task in a virtual reality environment

**DOI:** 10.14814/phy2.15025

**Published:** 2021-09-20

**Authors:** Frédérique Dupuis, Gisela Sole, Craig A. Wassinger, Hamish Osborne, Mathieu Beilmann, Catherine Mercier, Alexandre Campeau‐Lecours, Laurent J. Bouyer, Jean‐Sébastien Roy

**Affiliations:** ^1^ Faculty of Medicine Université Laval Quebec City Canada; ^2^ Centre for Interdisciplinary Research in Rehabilitation and Social Integration Quebec City Canada; ^3^ Centre for Health, Activity and Rehabilitation Research School of Physiotherapy University of Otago Dunedin New Zealand; ^4^ Physical Therapy Program East Tennessee State University Johnson City TN USA; ^5^ Department of Medicine Otago Medical School University of Otago Dunedin New Zealand; ^6^ Faculty of Science and Engineering Université Laval Quebec City Canada

**Keywords:** Experimental pain, kinematics, motor adaptations, shoulder

## Abstract

**Background:**

People with chronic shoulder pain have been shown to present with motor adaptations during arm movements. These adaptations may create abnormal physical stress on shoulder tendons and muscles. However, how and why these adaptations develop from the acute stage of pain is still not well‐understood.

**Objective:**

To investigate motor adaptations following acute experimental shoulder pain during upper limb reaching.

**Methods:**

Forty participants were assigned to the Control or Pain group. They completed a task consisting of reaching targets in a virtual reality environment at three time points: (1) baseline (both groups pain‐free), (2) experimental phase (Pain group experiencing acute shoulder pain induced by injecting hypertonic saline into subacromial space), and (3) Post experimental phase (both groups pain‐free). Electromyographic (EMG) activity, kinematics, and performance data were collected.

**Results:**

The Pain group showed altered movement planning and execution as shown by a significant increased delay to reach muscles EMG peak and a loss of accuracy, compared to controls that have decreased their mean delay to reach muscles peak and improved their movement speed through the phases. The Pain group also showed protective kinematic adaptations using less shoulder elevation and elbow flexion, which persisted when they no longer felt the experimental pain.

**Conclusion:**

Acute experimental pain altered movement planning and execution, which affected task performance. Kinematic data also suggest that such adaptations may persist over time, which could explain those observed in chronic pain populations.

## Graphical abstract

Acute experimental pain alters movement planning and execution. Kinematic data suggest that motor adaptations may persist over time, which could explain those observed in chronic pain populations.

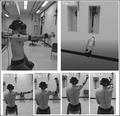

## 1 INTRODUCTION

People suffering from chronic shoulder pain such as rotator cuff related shoulder pain (RCRSP) often present with motor adaptations during arm movements (Bachasson et al., 2015; Chester et al., 2010; Hodges, 2011; Ludewig & Cook, 2000; Tomita et al., 2017). Examples of such adaptations include alterations in kinematics, evidenced with increased sternoclavicular (SC) elevation, decreased scapulothoracic upward rotation (Ludewig & Cook, 2000) and decreased glenohumeral (GH) elevation during arm movements (Hodges & Tucker, 2011; Lewis et al., 2015). Other examples of motor adaptations include changes in electromyographic (EMG) activity (e.g., reduced middle deltoid and rotator cuff activity and increased upper trapezius activity), and delayed muscular recruitment (e.g., delay in the onset of EMG activation of the upper trapezius and serratus anterior; Chester et al., 2010; Kinsella et al., 2017).

Several theories have been proposed to explain such adaptations, including the theory of a protective response to pain: motor adaptations following pain may initially aim to protect the system against the painful stimulus (Merkle et al., 2020). However, in the long term, these adaptations may lead to maladapted motor patterns and to abnormal physical stress on tendons and muscles (Hodges, 2011; Hodges & Tucker, 2011; Lefevre‐Colau et al., 2018; Lewis et al., 2015; Ludewig & Braman, 2011; Ludewig & Cook, 2000), likely contributing to the chronicity of pain (Lefevre‐Colau et al., 2018; Ludewig & Braman, 2011; Ludewig & Cook, 2000; Lukasiewicz et al., 1999). While most studies have investigated the presence of motor adaptations in chronic pain populations, adaptations following an acute onset of pain have been given little attention (Merkle et al., 2020). There is a need to study how motor adaptations develop during the acute phase of shoulder pain in order to better understand the underlying mechanisms leading to long‐term motor adaptations and persistence of pain.

Hypertonic saline injection at the shoulder has been used to evaluate the effect of acute experimental pain as it creates muscular or subacromial pain somewhat similar to the pain felt by individuals with RCRSP Bandholm et al., 2008; Madeleine et al., 1999, 2008; Sole et al., 2014, 2015; Stackhouse et al., 2013; Wassinger et al., 2012. To date, most of the studies that investigated the effect of acute experimental shoulder pain on motor adaptation (i.e., infraspinatus or upper trapezius intramuscular injection or injection in the subacromial space) have focused on muscle strength and EMG activity. Changes such as decreased strength in glenohumeral external and internal rotation, (Stackhouse et al., 2013; Wassinger et al., 2012) decreased abduction force steadiness, (Bandholm et al., 2008) and reorganization of muscles synergy (Madeleine et al., 1999; Sole et al., 2014) have been observed. While evidence supports that acute pain alters strength and EMG activity, fewer studies have investigated adaptations in kinematics and movement performance. Those who did, found that experimental shoulder pain lead to increased arm and trunk range of motion, increased movement variability, (Madeleine et al., 1999, 2008) reduced speed during a work‐related task, (Madeleine et al., 1999) and decreased throwing accuracy (Wassinger et al., 2012).

Most studies that have assessed adaptations to acute pain have used standardized tasks performed at less than 60° of arm elevation (Bandholm et al., 2008; Madeleine et al., 1999, 2008; Sole et al., 2014, 2015; Stackhouse et al., 2013; Wassinger et al., 2012). Performing tasks in these positions are known to be less challenging than performing overhead tasks (>60°). As shoulder stability is reduced in elevated positions, overhead activities are particularly demanding for the shoulder muscles, which is thought to increase the risk of developing motor adaptations potentially deleterious to the shoulder soft tissues (Rijn et al., 2010; Veeger & Helm, 2007). Motor adaptations during sustained overhead activities are highly prevalent among individuals with chronic RCRSP (Lewis et al., 2015; Ludewig & Cook, 2000; Lukasiewicz et al., 1999), showing the need to better understand how these adaptations develop following acute pain.

This study aimed to investigate motor adaptations to acute experimental shoulder pain during an elevated reaching task in a virtual reality environment (VRE). VRE was used to allow individualized positioning of the arm in a 3D space, while allowing targets to appear in unpredictable (randomized) order. EMG activity of the main agonists, upper limb and trunk kinematics and spatiotemporal data (i.e., movement performance) were collected to measure motor adaptations to pain. It was hypothesized that acute experimental pain would lead to:

1. Changes in muscle recruitment which will be evidenced by a decrease in the EMG activity of the scapulohumeral muscles activity such as the deltoid (Sole et al., 2014; Stackhouse et al., 2013; Wassinger et al., 2012), and an increase in the EMG activity of proximal muscles such as the upper trapezius (Madeleine et al., 1999; Wassinger et al., 2012).
2. Alterations in inter‐joint coordination (i.e., coordination between two or more joints when performing a movement Tomita et al., 2017), including greater use of proximal joints (sternoclavicular and trunk) and lesser use of distal joints (shoulder, elbow Chester et al., 2010; Hodges, 2011; Kinsella et al., 2017; Madeleine et al., 1999).
3. Decreased movement performance, including reduction of movement accuracy, movement velocity and reaction time, reflecting the cognitive costs of pain (Terrier & Forestier, 2009; Wassinger et al., 2012).

## 2 METHODS

### 2.1 Participants

This experimental study was conducted at the University of Otago, and *Université Laval*. The study population consisted of healthy young adults aged between 18 and 35 years with normal shoulder and neck motion, and no self‐reported upper limb or neck pain and disability. Participants were excluded if they had: (1) previous neck and upper limb surgery or fracture or (2) a history of glenohumeral dislocation (<12 months). Participants were recruited through the institutional mailing lists of the University of Otago and *Université Laval*, social medias, and posters around University of Otago's campus. Forty healthy adults, recruited in both New Zealand and Canada, took part in one laboratory session and were assigned to either the Pain group (the participants recruited in New Zealand; 20 participants, 10 men and 10 women) or the Control group (the participants recruited in Canada; 20 participants, 10 men and 10 women). For technical reasons related to the pain induction protocol, all participants that received pain were tested at the University of Otago. However, all data were collected by the same researcher, using the same experimental device and analyzed using identical procedures. The Sectorial Rehabilitation and Social Integration Research Ethics Committee of the *Centre intégré universitaire de santé et de services sociaux de la Capitale*‐*Nationale* (CIUSSS‐CN) and the University of Otago Human Ethics Committee approved this study and all subjects provided informed written consent.

### 2.2 Experimental procedure

Participants first completed a questionnaire on sociodemographics and the Edinburgh Handedness Inventory to establish hand dominance. Participants were also asked to rate the physical demands of their sports and work at the upper limbs on a scale of 0 to 100 (0 representing no physical requirement and 100 a maximum physical requirement). To familiarize themselves with the reaching task, they then completed a practice trial in the VRE. Finally, the participants completed the three phases of the experiment with their dominant arm:

- Baseline (BSL) phase: Participants performed the reaching task in the VRE.
- Experimental (EXP) phase: Prior to this phase, participants in the Pain group received the injection of hypertonic saline into the subacromial space and waited for two minutes. Participants in the Control group did not receive any injection but had a 5‐min break before completing the task again (time break similar to the average time it took to perform the injection protocol). Both groups then completed the same reaching task as in the Baseline phase.
- Post‐experimental (Post‐EXP) phase: Prior to this phase, participants in the Pain group waited until their pain was rated 0/10, while participants in the Control group had a 10‐min break (similar to the average time it took for the pain to be gone in the Pain group). Both groups then completed the same reaching task as in the previous phases.

During the baseline, EXP and post‐EXP phases of the experiment, EMG activity, upper limb and trunk kinematic and spatiotemporal data were collected.

### 2.3 Experimental pain

Experimental pain was induced for the participants in the Pain group using a single bolus injection of hypertonic saline (2.0 ml, 3% NaCl) into the subacromial space via a posterior approach by an experienced sports medicine physician Stackhouse et al., 2013. This saline concentration was chosen to create an intensity of pain similar to the one described by individuals with RCRP in their acute phase, aiming approximately 5/10 on a numeric pain rating scale (NPRS) Dupuis et al., 2018; Sole et al., 2015; Stackhouse et al., 2013; Wassinger et al., 2012. The effect of such an injection usually peaks 2 min after the injection (Stackhouse et al., 2013; Tsao et al., 2010), which was when the participants started the EXP phase. Pain lasted between 8 and 12 min, (Stackhouse et al., 2013; Tsao et al., 2010) which was long enough to perform the reaching tasks (which lasted less than a minute). Participants were asked to rate their perceived level of pain using the NPRS immediately after the injection, just prior to the EXP Phase, and right after the EXP Phase. They were also asked to describe the type of pain felt and its area.

### 2.4 Reaching task

Participants performed the reaching task in a VRE created in Unreal Engine (Epic games international, Unreal Engine) by wearing HTC VIVE goggles with 3D depth information (HTC corporation, VIVEPORT). They held a controller in their dominant hand that appeared to them as a virtual hand in the VRE (Figure 1.2). The task consisted of a series of four virtual targets (5 cm radius balls) that participants had to reach from a standardized initial position with their virtual hand (Figure 1.1). The use of the VRE allowed the targets to be placed around the participants relative to their anthropometric characteristics (arm length, height, etc.).

**FIGURE 1:**
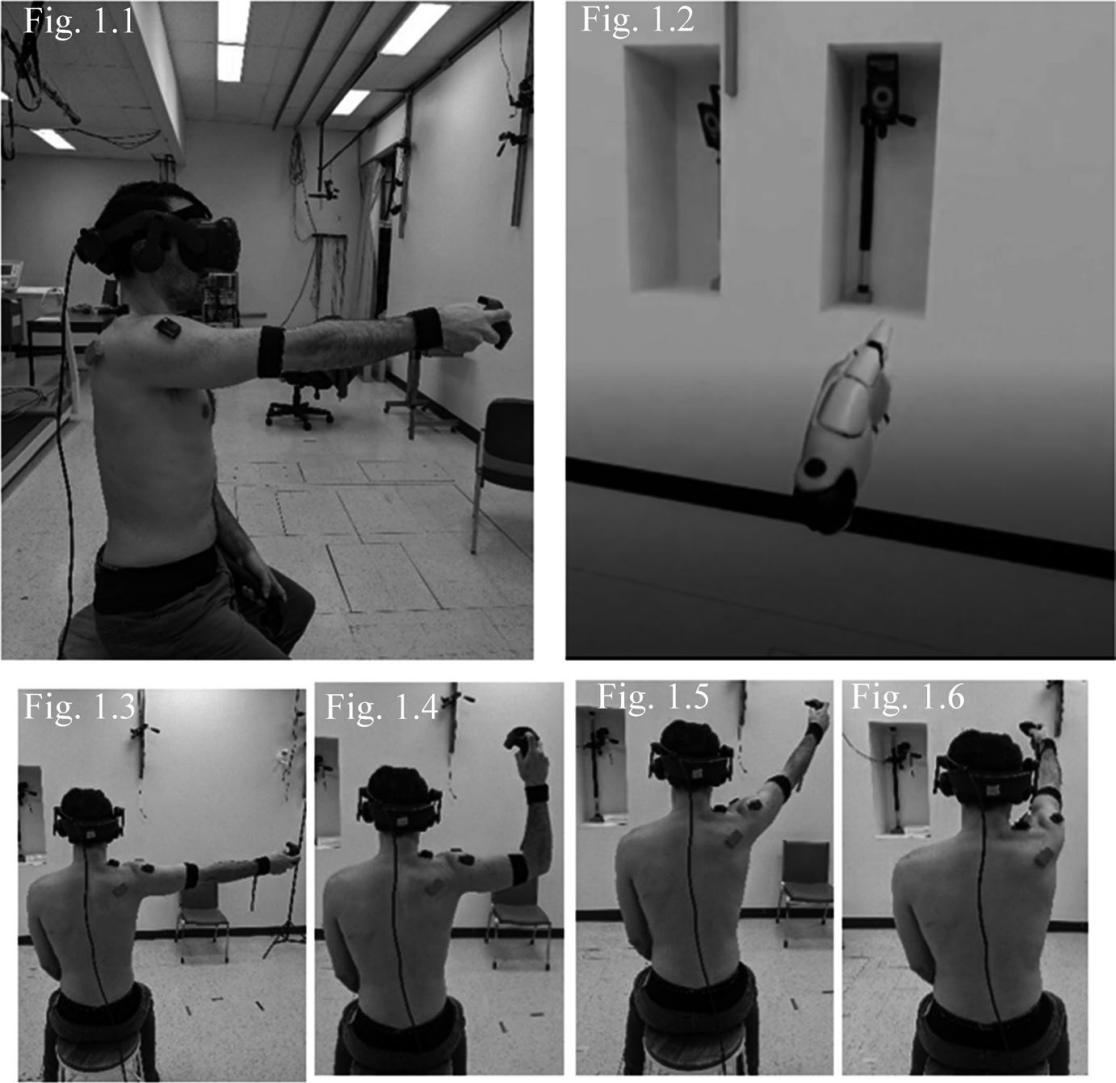
Experimental set‐up; (1.1) Initial position at 90 degrees of shoulder flexion; (1.2) virtual hand as seen by the participant in the VRE; (1.3) Target 1 at 90° of humeral abduction (ABD), elbow extended; (1.4) Target 2 at 90° humeral ABD + 90° external rotation (ER), 90° flexed elbow; (1.5) Target 3 at 120° of humeral elevation in the scapular plane, extended elbow, neutral humeral rotation and (1.6) Target 4 at 120° of humeral elevation in the sagittal plane (pure flexion), extended elbow, neutral humeral rotation

Participants performed the task seated, and their position was standardized at the beginning of each phase: straight back, knees flexed at 90° and feet on the floor. The targets’ positions were then defined for each participant in the VRE, confirmed with an electronic inclinometer (Figure 1.3–1.6):

Initial position's target (IPT): 90° shoulder flexion with the elbow fully extended and neutral humeral rotation
Target 1: 90° humeral abduction (ABD), elbow extended.
Target 2: humeral ABD +90° external rotation (ER), 90° flexed elbow.
Target 3: 120° humeral elevation in the scapular plane, extended elbow, neutral humeral rotation.
Target 4: 120° humeral elevation in the sagittal plane (pure flexion), extended elbow, neutral humeral rotation.

The participants were then familiarized with the reaching task in the VRE. When reaching the targets, they were instructed to place a 3‐cm virtual ball visible to them in the center of their virtual palm directly in the 5‐cm targets. The four above targets were randomly used five times per trial during each phase, thus a total of 20 consecutive targets. When reached by the participant's virtual hand, the target disappeared, and participants then had to return and stay on the IPT for 2 s to release the next target. Participants were instructed to reach the targets as accurately and as fast as possible. The trial ended when the participant reached the 20 targets.

This task was chosen for its requirements in terms of precision and speed, as well as its three‐dimensional aspect. To control for the potential influence of perceived level of exertion during the task, participants of both groups were asked to rate their perceived upper limb exertion level before and immediately after the reaching task using the Borg Rating of Perceived Exertion Scale (Borg CR10 Scale). They also had to rate their perceived level of pain immediately before and after the trials using the NPRS.

### 2.5 Instrumentation and data analysis

#### 2.5.1 Electromyographic activity

Wireless surface EMG sensors (Delsys Trigno) were placed on the anterior deltoid, middle deltoid, and upper trapezius of the dominant arm (Johnson et al., 2011; Poitras, Bielmann, et al., 2019). Anterior and middle deltoid muscles were chosen for their role as the main agonists for shoulder elevation, while upper trapezius for its role as the main agonist in sternoclavicular elevation. The anterior deltoid sensor was placed 1–2 cm below the acromioclavicular joint, the middle deltoid sensor midway of deltoid insertion and acromion and the upper trapezius sensor midway of C7 and acromion, according to the Surface EMG for Noninvasive Assessment of Muscles (SENIAM) guidelines (Hermens et al., 1999). The EMG activity was recorded using Delsys EMGworks^®^ Acquisition software (sampling rate: 1925.93 Hz).

To characterize muscle activity, EMG peak amplitude and mean area under the curve of the Root mean squared (RMS) processed EMG were extracted for each target and each evaluated muscle. Mean time to reach the peak amplitude was also computed to characterize the EMG activity curve and the EMG activity delay of muscle contraction (Konrad, 2005). To obtain these variables, all EMG signals were processed using custom software written in MATLAB R2013a (The MathWorks Inc.). EMG signals were digitally filtered off‐line with a zero‐lag 4^th^ order Butterworth Filter (band‐pass 20–450 Hz).

#### 2.5.2 Upper limb and trunk kinematics

Six inertial measurement units (IMUs) (MVN, Xsens Technologies; sampling rate: 60 Hz) were used to characterize upper limb and trunk kinematics during the task (Poitras, Dupuis, et al., 2019). IMUs were placed in accordance with Xsens suggested sensors configuration on the head, sternum, pelvis, scapula, upper arm, and forearm of the participant's dominant arm. The calibration consisted of a N‐Pose (arms alongside the body) followed by a slow 90° flexion of the arms and a slow anterior flexion of the trunk.

The acceleration and gyroscope data were then imported into MATLAB R2018a (The Math Works Inc.). Data fusion with a custom algorithm was performed to obtain the 3D orientation of each sensor (Boyer et al., 2020). Joint angles were then calculated relative to the orientation of the trunk and upper arm sensors. The tilt‐and‐torsion “TT‐Z” rotation sequence (similar to YZY) was also used to calculate Euler angles to obtain the arm elevation (second angle of TT‐Z and YZY; Campeau‐Lecours et al., 2020). The variable of interest was the mean total joint excursion for each joint (final angle – initial angle) during the reaching movements, calculated for each reached target. The initial angles were calculated while the participants were on the IPT (while waiting for the next target to be released, just before the reaching movement began). The final angle was calculated when the target was reached. The joints analyzed were the trunk (flexion/extension, lateral flexion, and rotation), sternoclavicular joint (elevation), elbow (flexion/extension), and glenohumeral joint (elevation, plane of elevation and rotation). Trunk lateral flexion, rotation, and the glenohumeral plane of movement were assessed when reaching toward targets 1, 2, and 3; glenohumeral rotation was assessed for movements toward target 2; all remaining movements were assessed across all the four targets.

#### 2.5.3 Spatiotemporal performance

Spatiotemporal data were collected with the controller held by the participants and by Unreal Engine software (sampling rate: 90 Hz; Niehorster et al., 2017). The variables used to characterize task performance were: (1) Reaction time, reflecting the delay in movement initiation (i.e., time between the moment the target was released and the moment the participant initiated the reaching movement [i.e., the moment the hand quit the ITP]) (2) Movement speed (i.e., time between the moment the participant initiated the reaching movement [i.e., the moment the hand quit the ITP] and the moment the target was reached); (3) the initial angle of endpoint deviation (iANG) which reflects movement planning as it was based on the initial trajectory of the hand (this angle was calculated using the shortest line between two targets [IPT and reaching targets] and the line corresponding to the initial peak of acceleration); (4) the final error (fERR) which reflects the accuracy of the movement (the shortest arc distance between the ideal arrival point into the target and the actual arrival point); and (5) the area under the curve representing total movement error (the summation of the rectangular trapezoids perpendicular to the ideal trajectory line and the actual trajectory line) Dupuis et al., 2021. Spatiotemporal data were extracted for each reaching movement using a custom software written in MATLAB. Mean values for each target were calculated and used for the analysis.

The three systems (Xsens, EMG and Unreal) were time‐synchronized using a custom trigger box.

### 2.6 Statistical analysis

Baseline demographic data were compared between groups using independent *t*‐tests and χ2. For the perceived level of exertion, EMG outcomes, kinematics and spatiotemporal data, a three‐way repeated measures ANOVA was used to calculate the effect of Time (BSL, EXP, Post‐EXP phases), Group (Pain group, Control group), and Targets when applicable (1, 2, 3, and 4). Only the interaction between Time x Group was considered. Inherent post‐hoc tests were conducted to detail interactions between factors. All statistical tests were conducted in IBM SPSS Statistics (IBM SPSS Statistics 26, IBM Corp., NY, USA) with a significance level set at 0.05.

## 3 RESULTS

Table 1 presents their baseline characteristics; there were no significant differences between the groups (*p* > 0.05).

**TABLE 1:** Participants’ characteristics and perceive level of exertion during the task

Characteristics	Pain Group *n* = 20	Control Group *n* = 20
Gender, female *n*, %	10 (50)	10 (50)
Height, cm	172.5 (12.4)	173.2 (12.3)
Weight, kg	71.8 (14.9)	74.4 (15.8)
Age, years	26.6 (3.8)	26.1 (3.2)
Dominance, right *n*, %	19 (95)	20 (100)
Sports^*^, %	51.6 (37.1)	52.0 (31.3)
Work^*^, %	42.3 (42.0)	37.8 (31.8)
Perceived level of exertion		
Borg score BSL	2.4 ± 1.0^*^	2.6 ± 1.0
Borg score EXP	4.9 ± 1.0^a^^,^ ^*^	2.7 ± 1.3
Borg score Post‐EXP	2.7 ± 1.0	2.6 ± 1.7

Note

Data are presented as mean (SD). No significant difference between the groups for all characteristics (independent *t*‐test and χ2, *p* > 0.05).

Abbreviations: Borg score, Perceived level of exertion on the Borg rating scale (0–10); BSL, Baseline phase; EXP, Experimental phase; Post‐EXP, Post experimental phase.

^a^ Participants were asked to rate the physical demands of their sports and work at the upper limbs (0 = no physical demands, 100 = maximal physical demands).

* Significant Time × Group interaction (*p* < 0.001), Post‐hoc analysis showed a significant difference between the baseline phase mean Borg score and the experimental phase mean Borg score of the Pain group (*p* < 0.001).

### 3.1 Experimental pain

All participants in the Pain group reported a level of pain greater than 3 on the NPRS after the injection. Mean perceived level of pain was 5.9 ± 1.7 right after the injection, 4.6 ± 2.4 just before the beginning of the EXP Phase (mean time 2.5 ± 0.1 min after the injection), and 3.3 ± 1.3 immediately after the EXP Phase (mean time 4.2 ± 0.1 min after the injection). It took an average of 11.5 ± 0.2 min after the injection before the participants in the Pain group no longer felt any pain (0/10) and had full pain free shoulder range of motion. Participants in the Pain group reported no longer feeling pain (0/10) and had full pain free shoulder range of motion, on average, 11.5 ± 0.2 min after the injection.

The pain perception was reported as ‘deep’ and ‘achy’ by ten participants and was also compared to ‘a punch in the arm’ by eight participants. The most reported areas for pain after the injection were the posterior and lateral subacromial areas, reported by 16 participants, and seven participants also felt radiating pain toward the lateral deltoid area. The participants in the Control group did not report any pain during the experiment.

### 3.2 Perceived level of exertion

There was a significant impact of the experimental pain on the perceived level of exertion during the task (Time × Group interaction, *p *< 0.001, Table 1). At baseline, there was no difference for the mean perceived level of exertion between the groups after the completion of the task (*p *= 0.367), but there was significant difference after completing the EXP phase (*p *< 0.001). The Experimental Pain group perceived the task more demanding when they were in pain (*p *< 0.001), while the Control group maintained a similar level between baseline and EXP phase (*p *= 0.346). During the Post‐EXP phase, the Pain group returned to a similar level of exertion compared to baseline and there was no significant difference between the groups at this stage.

### 3.3 Electromyographic activity

There was a statistically significant Time × Group interaction for the time to reach the peak amplitude for the anterior deltoid and upper trapezius (*p *< 0.001) and for the area under the curve of the upper trapezius (*p *< 0.001).

**FIGURE 2:**
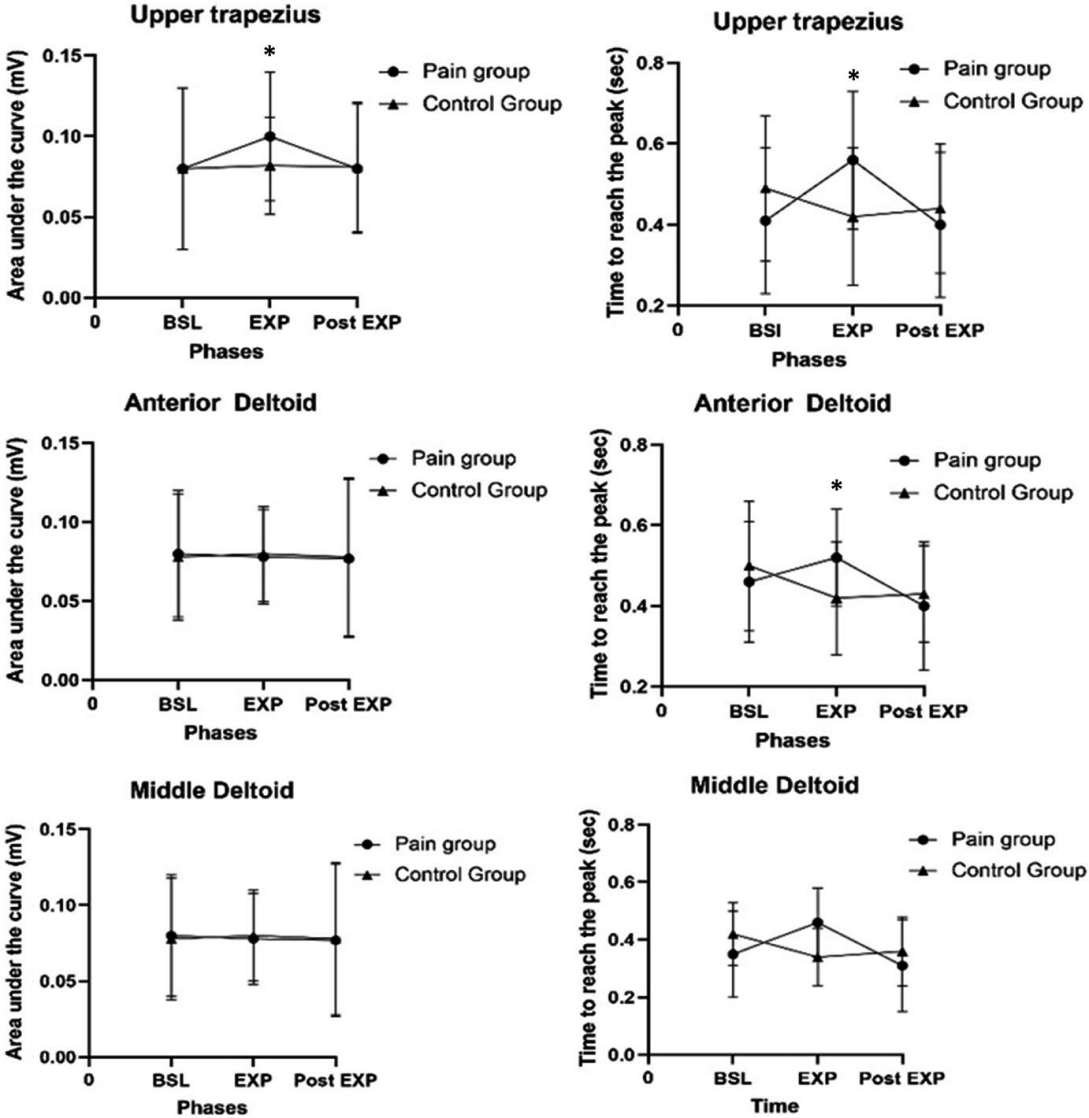
Electromyographic activity during the task. BSL, baseline; Exp, experimental phase; Post‐Exp, post‐experimental phase; SD, Standard deviation. Results are presented as mean ± SD values while reaching the four targets. *Significant Time x Group interaction for the time to reach the peak amplitude for the anterior deltoid and upper trapezius (*p* < 0.001) and for the area under the curve of the upper trapezius (*p* < 0.001)

Post hoc analyses showed that the presence of pain slowed muscles recruitment (upper trapezius and anterior deltoid): participants in the Pain group had an increase in time to reach the peak during the EXP phase compared to baseline for the anterior deltoid (*p *= 0.018) and upper trapezius, although not significant (*p *= 0.062), while the Control group showed a decrease in time to reach the peak for both muscles through the phases compared to baseline (EXP Phase, *p *< 0.001; Post‐EXP phase, *p *= 0.001, Figure 2). In the Post‐EXP phase, the Pain group showed a reduction of the mean time to peak for the anterior deltoid and the upper trapezius compared to baseline (*p *= 0.071 and *p *= 0.008 respectively).

A significant increase in the upper trapezius total area under the curve occurred during the EXP phase for the participant in the Pain group (Baseline vs. EXP phase, *p *= 0.020, Figure 2), which then returned close to baseline values during the Post‐EXP phase (Baseline vs. Post‐EXP phase, *p *= 0.936). The control group did not show changes between the phases and there were no other significant differences for the other EMG variables.

### 3.4 Kinematic data

**FIGURE 3:**
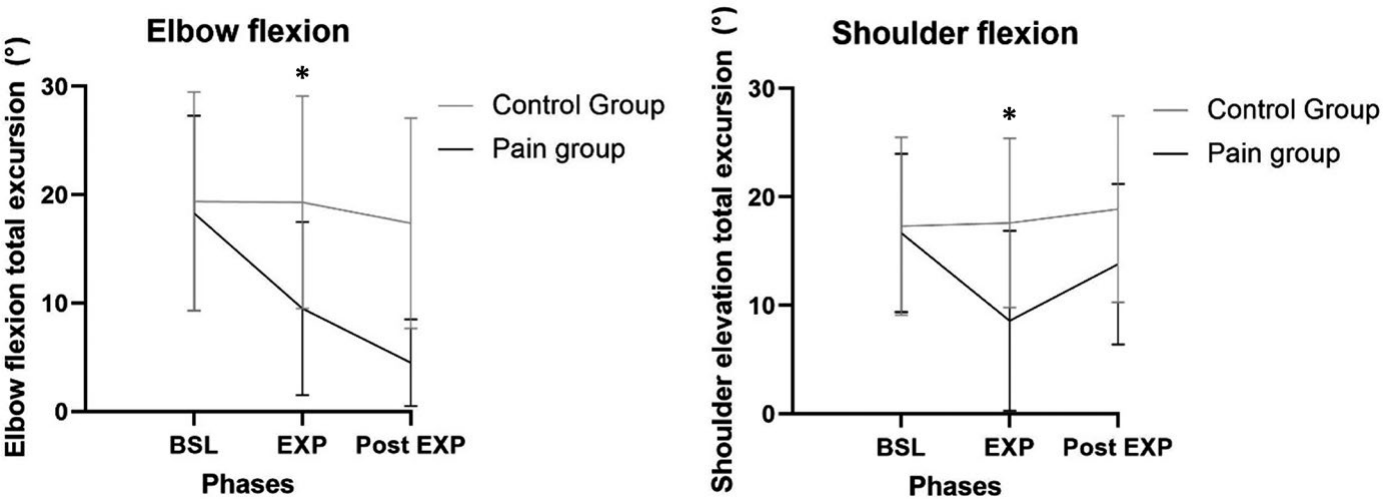
Upper limb kinematics. BSL, baseline; EXP, experimental; Post EXP, Post‐experimental; SD, Standard deviation. Results are presented as mean ± SD values while reaching the four targets. *Significant Time x Group interaction for elbow and shoulder total excursion. Pain group significantly reduced their shoulder and elbow total excursion during the EXP and post EXP phases (*p* < 0.05)

There was a significant Time x Group interaction in total excursion for glenohumeral elevation (*p *= 0.028, Figure 3), glenohumeral plane of movement (*p *= 0.012), trunk lateral flexion (*p*= 0.023), and elbow flexion (*p *= 0.013, Figure 3).

The Pain group used a different inter‐joint coordination during the EXP phase compared to baseline. They used less shoulder elevation (*p* = 0.015) and elbow flexion (*p* = 0.006). These kinematics adaptations were maintained during the Post‐EXP phase (Baseline vs. Post‐EXP phases, *p* = 0.05 and *p* = 0.04, respectively), during which an increase in trunk contro‐lateral flexion occurred (Baseline vs. Post‐EXP phase, *p *< 0.001). The Control group did not show such inter‐joint coordination changes between the phases (*p* > 0.05). However, participants in the Control group significantly changed their shoulder plane of movement through the phases to complete the task, as reaching was performed more in the frontal plane rather than the sagital plane (Baseline vs. EXP phase, *p* < 0.001; Baseline vs. Post‐EXP phase, *p* = 0.026). Participants in the Pain group did not show such change.

### 3.5 Spatiotemporal data

**FIGURE 4:**
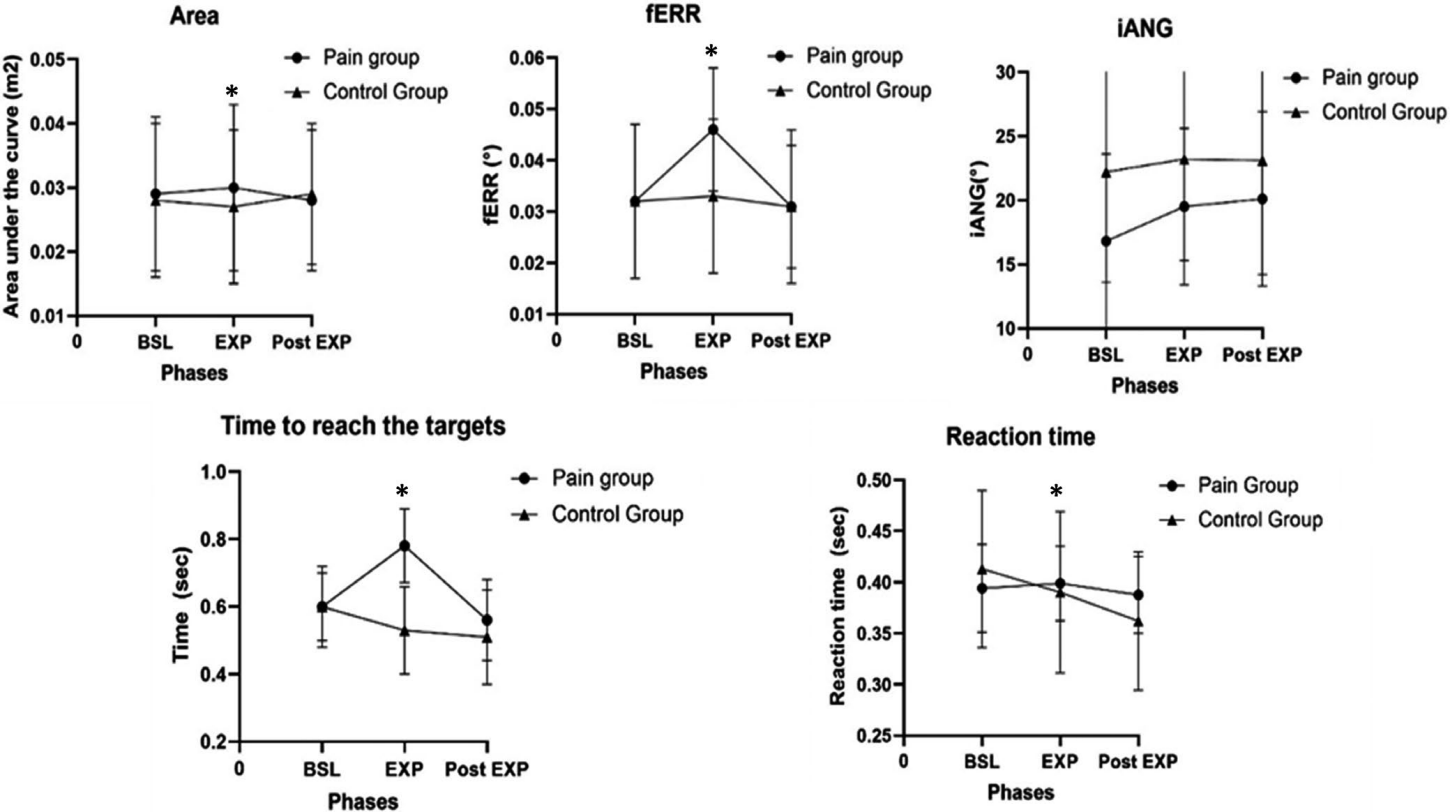
Performance results. BSL, Baseline; EXP, experimental phase; Post EXP, post‐experimental phase; Area, area under the curve; fERR, final error; iANG, initial angle; Time, time to reach the peak; SD, Standard deviation. Data are presented as mean ± SD values for the four targets. *There was a significant Time x Group interaction for the fERR (*p* = 0.037), area under the curve (*p* = 0.047), time to reach the target (*p* = 0.011) and Reaction time (*p* = 0.047)

There was a significant Time × Group interaction for reaction time (*p* = 0.041), movement speed (*p* = 0.01), fERR (*p* = 0.037), and area under the curve (*p *= 0.047, Figure 4).

Participants in the Control group showed a decrease in their mean reaction time through the phases. They showed faster movement initiation during EXP and Post‐EXP phases compared to baseline (*p* = 0.001), while participants in the Pain group did not show any changes compared to baseline (EXP and Post‐EXP phases, *p* = 0.169). As for movement speed, post hoc analysis showed that the Pain group did not show a significant reduction in their mean time to reach the targets during the EXP phase compared to Baseline (*p* = 0.137), while the Control group did (Baseline vs. EXP phase, *p* = 0.001; Baseline vs. Post‐EXP phase, *p* < 0.001). However, when they no longer had pain, the Pain group improved their speed compared to baseline (Baseline vs. Post‐EXP phase, *p* = 0.012).

Participants in the Pain group increased their mean final error during the pain condition compared to baseline (EXP phase, *p* = 0.049) and returned close to their baseline values during the Post‐EXP phase (*p* = 0.793). The Control group did not show any changes between the phases compared to baseline (*p* = 0.893).

Post hoc analysis did not identify any changes between the phases for both groups’ mean area under the curve (*p* = 0.085). There was also no Time × Group interaction for the iANG of endpoint deviation (*p* = 0.070).

## 4 DISCUSSION

We investigated motor adaptations to acute experimental shoulder pain during an upper limb reaching task in an elevated arm position and in a VRE. The Pain group showed significant motor adaptations with pain, including reduction in shoulder and elbow movements, delayed EMG peak activity, increased upper trapezius activity, and reduction of movement accuracy. In contrast, the Control group showed changes across the experimental phases that were mostly related to improved performance, such as faster muscle recruitments, faster movement initiations, and faster reaching movements.

Changes in EMG activity are slightly different from what was hypothesized. As expected, the Pain group showed an increase in the EMG activity the upper trapezius, the main sternoclavicular elevator. However, we also expected to see a reduction in glenohumeral elevator muscles (i.e., deltoids) activity, which did not occur, even though less shoulder elevation occurred for the pain group during the EXP phase (Falla et al., 2007; Lund et al., 1991; Nussbaum et al., 2001; Stackhouse et al., 2013). These results may be explained by changes in central motor planning aiming for protective mechanism to achieve pain‐free movement Hodges, 2011. These changes likely reduced mechanical stress on the painful subacromial structures (Hodges & Tucker, 2011). The increase of the upper trapezius activity could have compensated for this shoulder elevation reduction by increasing sternoclavicular elevation Ludewig & Braman, 2011; Nussbaum et al., 2001. The lack of significant changes in scapular elevation could be explained by the great variability among participants in the amount of sternoclavicular elevation used during the task (i.e., large SD).

Muscle activity was characterized with mean time to reach EMG peak activity, describing EMG activity curve and defining mean time to reach muscle contraction peak (Konrad, 2005). Deltoid peak recruitment was delayed in the pain group during the EXP phase, while the control group showed a faster peak recruitment for the anterior deltoid and upper trapezius through the phases. These EMG changes appeared to be related to the reaction time: the Control group reduced their mean time before initiating the reaching movement in the EXP phase while the Pain group did not. Experimental pain has been shown to affect reaction times, measured either by movement initiation or muscle recruitment delay. While studies that used constant pain (i.e., hypertonic saline injection Ervilha et al., 2004a; Ervilha et al., 2004b; Madeleine et al., 1999) or acute pain related to movement initiation (Neige et al., 2018) showed increased reaction times, studies that used painful stimuli prior to movement onset have instead showed shorter reaction times (Misra et al., 2017; Perini et al., 2013). It appears that when movement initiation reduces the painful stimulus, pain does not affect movement planning since reaction times are reduced. In contrast, movement planning seems to be negatively affected when apprehending more pain, similar to the pain experienced by people with musculoskeletal disorders. This could be explained by the fear of pain that could reduce the attention of participants Bouffard et al., 2018; Lamothe et al., 2014; Mercier et al., 2016. Indeed, divided attention has been shown to impair movement planning (Taylor & Thoroughman, 2007). The mechanisms underlying reduced attention with pain remains uncertain, but we have previously demonstrated similar results during a virtual reaching task in a fatigue state (Dupuis et al., 2021). Cognitive or physiological costs of exploring protective motor patterns at the CNS in response to a new body state could be involved. (Dupuis et al., 2021; Eccleston & Crombez, 1999; Nederhand et al., 2006; Vuillerme & Pinsault, 2009; Wassinger et al., 2012).

The Pain group did not improve their reaching speed like the Control group did and showed an increase of their mean final error (fERR). It is thus reasonable to assume that the presence of pain in the present study led to alterations in motor execution. Previous findings suggest that performance can be maintained despite the presence of pain through the adoption of different motor strategies, for example, during isometric pinch task Mercier et al., 2016 and locomotor adaptations task (Bouffard et al., 2018). However, others have observed increased final error while reaching targets against a force field with cutaneous pain at the upper limb (Lamothe et al., 2014), as well as a reduction of throwing accuracy following sub‐acromial hypertonic saline injection (Wassinger et al., 2012). Thus, as distinct types of experimental pain can affect movement planning and execution in different ways. The tasks requirements may also affect the degree of the observed alterations. The performance of a task with higher demands, for example requiring higher speed and accuracy, will be affected to a greater extent in the presence of pain‐altered movement planning and motor execution (Kawato, 1999; Lamothe et al., 2014). This highlights the importance of taking into account the type of pain experienced by people with pain as well as the physical demands of their daily living activities in order to understand the impact pain may have on their motor performance.

While most motor adaptations for the Pain group returned close to baseline values during the Post‐EXP phase (e.g., EMG activity, reaching speed, and accuracy), some kinematic adaptations persisted, such as decreased excursion at the elbow and increased contra‐lateral lateral flexion at the trunk. As previously mentioned, pain adaptation theories state that persistent motor adaptations may increase physical stress on the peri‐articular structures and contribute to chronic pain (Lefevre‐Colau et al., 2018; Ludewig & Braman, 2011; Ludewig & Cook, 2000; Lukasiewicz et al., 1999). Although the impact of these adaptations on peri‐articular structures remains uncertain, our results support the theory that the acquisition of altered motor patterns in pain may persist with the resolution of acute pain (Hodges & Tucker, 2011; Lamothe et al., 2014).

The Pain group also showed an increase of perceived level of exertion in the presence of pain. A clear link appears to exist between the perception of pain and perceived exertion, but the underlying mechanisms of acute pain on exertion perception are still not well‐understood (Louati & Berenbaum, 2015; Mense & Schiltenwolf, 2010; Sluka & Rasmussen, 2010). It is difficult to determine the extent to which the increased perceived level of exertion affected motor adaptations in this study, but it is well‐known that it leads to motor adaptations at the upper limb and decreases performance (Chopp et al., 2011;Chopp et al., 2010; Ebaugh et al., 2006; Forestier & Nougier, 1998; McDonald et al., 2019; McDonald et al., 2016). Interestingly, in the Post‐EXP phase, residual changes in kinematics were still evident despite the self‐perceived exertion having returned to baseline values.

Although we used a lower concentration of 3% NaCl than reported in previous studies, a definite pain experience was created. We suggest that the reported intensity, approximately 5/10 on the NPRS is similar to the intensity commonly reported for individuals with RCRSP. The pain was mostly described as deep in the deltoid area of the shoulder, which is close to the pain experienced by patients Sole et al., 2014. However, subacromial pain is usually reproduced by movement when peri‐articular and muscle‐tendinous structures are loaded Lewis et al., 2015; Roy et al., 2009. The experimentally induced pain did not reproduce this effect, as participants most frequently reported the pain to be constant, regardless of position or movement, until it subsided. Caution is thus needed when extrapolating the responses to the experimental pain to a population with acute clinical subacromial pain. Finally, although the two groups were tested in different laboratories in two different countries, two research team members lead data collection using the same equipment in both laboratories. The two participant groups were similar at baseline with respect to the EMG, kinematic, and performance variables, as well as to the participants’ characteristics, which reduces the potential bias related to the population in this study.

## 5 CONCLUSION

This study provides new knowledge on motor adaptations to acute experimental shoulder pain during a functional elevated task. When the participants were in pain, movement planning and execution were affected, resulting in delayed muscle recruitment and decreased accuracy. Protective motor adaptations were also objectified, including a reduced upper limb total movement and increased upper trapezius activity. The persistence of such kinematic adaptations even after the experimental pain disappeared could explain long‐term persistent residual motor adaptions observed in people with clinical chronic musculoskeletal pain.

## CONFLICT OF INTEREST

The authors have no conflicts of interest to report.

## AUTHOR CONTRIBUTION

JSR, GS, FD: Coordination of the study, design and writing of the protocol. FD, GS, CW, HO, JSR: Data collection. ACL, MB, LB: Development of the EMG and Kinematic data extraction software on MATLAB. FD: Data analysis, statistical analysis, manuscript write up. FD, JSR, GS, CW, HO, ACL, MB, LB, CM: Data interpretation, critical revision of the article.
